# Intracolic ultrasound molecular imaging: a novel method for assessing colonic tumor necrosis factor-α expression in inflammatory bowel disease

**DOI:** 10.1186/s10020-021-00379-z

**Published:** 2021-09-23

**Authors:** Xiaoyan Miao, Ren Mao, Yujia You, Huichao Zhou, Chen Qiu, Xuehua Li, Zhihui Chen, Jie Ren, Minhu Chen, Ping Wang, Rongqin Zheng, Tinghui Yin

**Affiliations:** 1 Department of Ultrasound, Laboratory of Novel Optoacoustic (Ultrasonic) Imaging, The Third Affiliated Hospital of Sun Yat-Sen University, Guangzhou, 510630 China; 2 Department of Gastroenterology, The First Affiliated Hospital of Sun Yat-Sen University, Guangzhou, 510120 China; 3 Department of Radiology, The First Affiliated Hospital of Sun Yat-Sen University, Guangzhou, 510120 China; 4 Department of Gastrointestinal Surgery, The First Affiliated Hospital of Sun Yat-Sen University, Guangzhou, 510120 China

**Keywords:** Intracolic ultrasound molecular imaging, Targeted microbubbles, Tumor necrosis factor alpha, Inflammatory bowel disease

## Abstract

**Background:**

While anti-tumor necrosis factor alpha (TNF-α) therapy has been proven effective in inflammatory bowel disease (IBD), approximately 40% of patients lose the response. Transmembrane TNF-α (mTNF-α) expression in the intestinal mucosa is correlated with therapeutic efficacy, and quantification of mTNF-α expression is significant for predicting response. However, conventional intravenous application of microbubbles is unable to assess mTNF-α expression in intestinal mucosa. Herein, we proposed intracolic ultrasound molecular imaging with TNF-α-targeted microbubbles (MB_TNF-α_) to quantitatively detect mTNF-α expression in the intestinal mucosa.

**Methods:**

MB_TNF-α_ was synthesized via a biotin–streptavidin bridging method. TNF-α-targeted ultrasound imaging was performed by intracolic application of MB_TNF-α_ to detect mTNF-α expression in surgical specimens from a murine model and patients with IBD. Linear regression analyses were performed to confirm the accuracy of quantitative targeted ultrasound imaging.

**Results:**

On quantitative TNF-α-targeted ultrasound images, a greater signal intensity was observed in the mouse colons with colitis ([1.96 ± 0.45] × 10^6^ a.u.) compared to that of the controls ([0.56 ± 0.21] × 10^6^ a.u., *P* < 0.001). Targeted US signal intensities and inflammatory lesions were topographically coupled in mouse colons. Linear regression analyses in specimens of mice and patients demonstrated significant correlations between the targeted ultrasound signal intensity and mTNF-α expression (both *P* < 0.001). Furthermore, TNF-α-targeted ultrasound imaging qualitatively distinguished the varying inflammatory severity in intestinal specimens from IBD patients.

**Conclusion:**

Intracolic ultrasound molecular imaging with MB_TNF-α_ enables quantitative assessment of mTNF-α expression. It may be a potential tool for facilitating the implementation of personalized medicine in IBD.

**Supplementary Information:**

The online version contains supplementary material available at 10.1186/s10020-021-00379-z.

## Introduction

Inflammatory bowel disease (IBD), including Crohn’s disease (CD) and ulcerative colitis (UC), has become a global disease with increasing incidence (Ng et al. 2018). It is widely accepted that the cytokine tumor necrosis factor alpha (TNF-α), which initiates and amplifies intestinal inflammation by activating multiple signaling pathways, plays a crucial role in the immunopathogenesis of IBD (Atreya et al. 2011). Anti-TNF therapies using monoclonal antibodies (such as infliximab and adalimumab) are effective for treating moderate to severe IBD (Lightner et al. 2017). Nevertheless, approximately 40% of patients fail such treatment due to primary or secondary nonresponse (West et al. 2017), which exposes them to potential side effects such as infection and allergy with little or no alleviation of clinical symptoms (Ben-Horin et al. 2014). Thus, predicting response to anti-TNF therapy is an important unmet clinical need.

Clinical and laboratory factors, including disease duration, disease phenotype, C-reactive protein, fecal calprotectin and lactoferrin, are commonly introduced before anti-TNF therapy but with low specificity for responsiveness prediction (Naviglio et al. 2018). Determining transmembrane TNF-α (mTNF-α) expression in the intestinal mucosa is another promising strategy (Atreya et al. 2014; Olsen et al. 2009). Emerging endoscopy techniques, such as confocal laser endomicroscopy (CLE), were used for in vivo detection of mTNF-α expression during colonoscopy and prediction of response to anti-TNF therapy in landmark clinical trials (Atreya et al. 2014; Kattah and Mahadevan 2015*).* However, CLE has limited vision, cannot access the full length of the colon and small bowel and easily loses focus (Rasmussen et al. 2015).

Ultrasonography, which is a noninvasive and real-time cross-sectional imaging modality (Whitman and Hortobagyi 2017), is able to provide full-length and transmural vision of the intestine and has been widely used to assess disease activity and treatment response (Panes et al. 2013; Gomollón et al. 2017). Molecular imaging has been hailed as a great advance for medical imaging, as it is capable of visualizing biological processes at the cellular and molecular levels (Hoffman and Gambhir 2007). Ultrasound molecular imaging (USMI) has been used to evaluate breast and ovarian cancer in a clinical trial using clinical-grade targeted microbubbles (MBs) (Willmann et al. 2017). In our previous study, we successfully performed USMI with targeted intracellular adhesion molecule 1 to quantitatively diagnose and monitor the therapeutic response of hepatic ischemia–reperfusion injury (Qiu et al. 2017). Moreover, USMI has been applied to assess active bowel inflammation in animal models of IBD (Wang et al. 2013; Tlaxca et al. 2013; Machtaler et al. 2015; Deshpande et al. 2012; Bachmann et al. 2006). However, all previous studies applied MBs intravenously, which did not allow penetration of the blood vessels to detect mTNF-α expression in the intestinal mucosa due to the microscale size of the MBs. Furthermore, to the best of our knowledge, few studies have reported on TNF-targeted or response prediction-oriented USMI in IBD.

Herein, we synthesized MBs targeting mTNF-α and assessed the feasibility of quantitatively detecting mTNF-α expression in the intestinal mucosa of a murine model of colitis by applying intracolic USMI. Furthermore, we applied this novel technique in surgically resected intestinal specimens from IBD patients, aiming to establish a strategy with potential application in predicting the response to anti-TNF therapy and assisting precision medicine in IBD.

## Materials and methods

### Preparation of targeted microbubbles

**Fig. 1:**
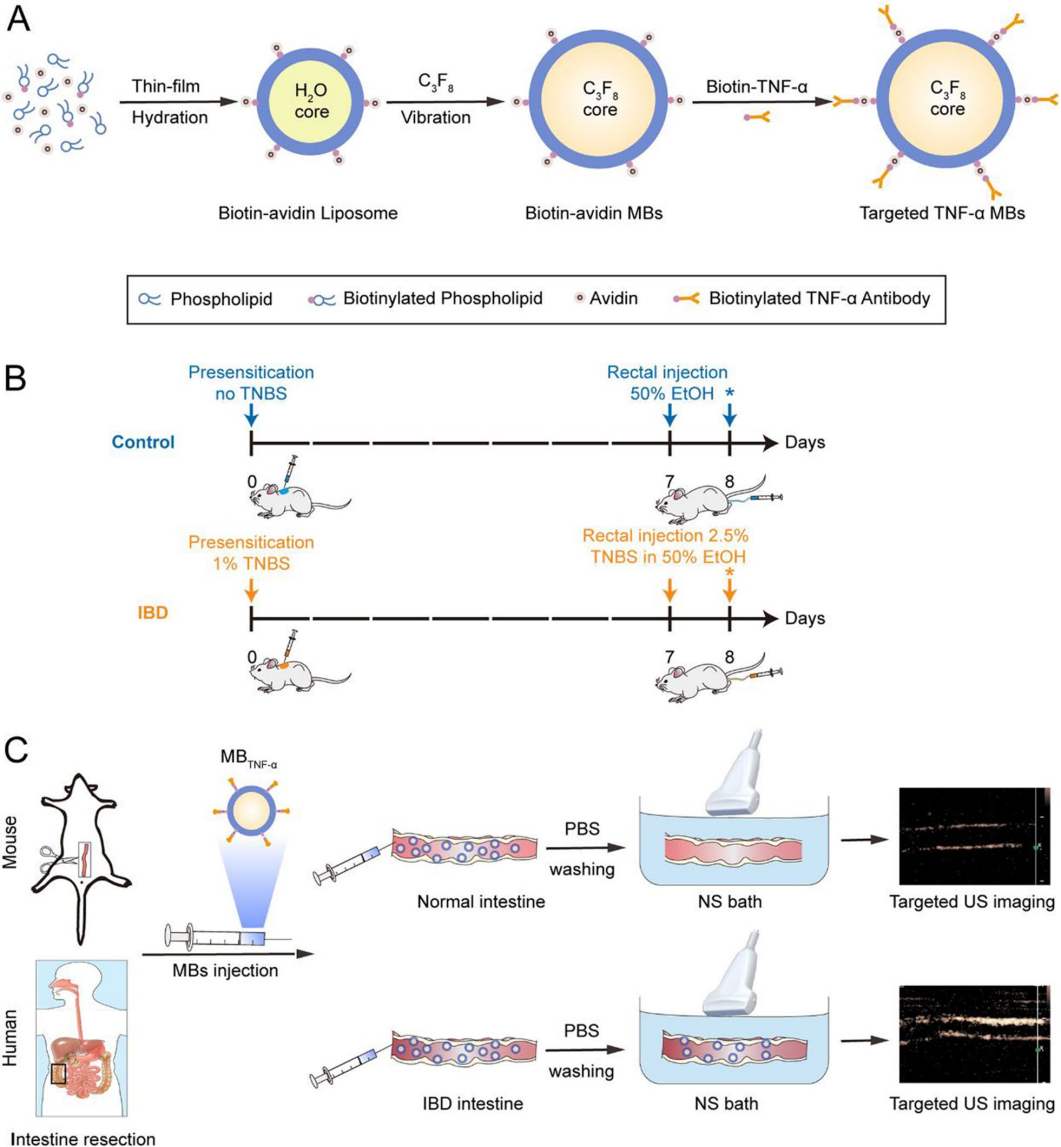
Schematics for the study. **A** Preparation of microbubbles (MBs). MBs were fabricated via a biotin–streptavidin bridging chemistry method. **B** Schematic for control and TNBS-treated mice including presensitization with or without TNBS at day 0, followed by rectal injection of either 50% ethanol (EtOH) or 2.5% TNBS in 50% EtOH at day 7, and all mice were sacrificed at day 8 (*). **C** Schematic for ex vivo ultrasound imaging of mice and human specimens. *IBD* inflammatory bowel disease, *TNBS* 2,4,6-trinitrobenzenesulfonic acid, *NS* normal saline

TNF-α-targeted MBs (MB_TNF-α_) and control MBs (MB_con_) were synthesized according to a multistep biotin–streptavidin bridging chemistry method modified from a previously reported study (Lindner et al. 2001) (Fig. 1A). The size and zeta potential of MB_TNF-α_ and MB_con_ were measured by dynamic light scattering (DLS) analysis. The specific conjunction of TNF-α antibody to the surface of MBs was determined by inverted fluorescence microscopy. The microbubble concentration was determined by using an automated cell counter (Bio-Rad, USA). The amount of antibody on the surface of microbubbles was measured by a spectrofluorometer (Tecan Spark, Austria) according to a previous report (Takalkar et al. 2004). All detailed procedures are described in the Additional file 1.

## Animal experiment

### Murine model of TNBS-induced acute colitis

All experimental procedures involving animals were in accordance with the Guide for the Care and Use of Laboratory Animals (NIH publication Nos. 80–23, revised 1996) and followed the institutional ethical guidelines for animal experiments. All procedures and animal care were approved by the local Ethics Committee of the Third Affiliated Hospital, Sun Yat-sen University. Acute colitis was induced in 6–8-week-old male BALB/c mice (Guangdong Medical Laboratory Animal Center, China) by rectal administration of 2,4,6-trinitrobenzenesulfonic acid (TNBS) in absolute ethanol (EtOH), while control mice were treated with 50% EtOH, as reported previously (Bruyn et al. 2017) (Fig. 1B and Additional file 1). The total number of mice enrolled in this study was 38. Among them, 20 mice were used to establish and validate the IBD model, and another 18 mice were used to perform *ex vivo* intracolic ultrasound imaging.

### Macroscopic and histological evaluation of colonic inflammation

Mice were euthanized with sodium pentobarbital. The disease activity index (DAI) score was determined according to loss of body weight (one point for each 5% loss of weight), consistency of stools (normal = 0, soft = 2, liquid = 4) and presence of gross blood in stool (0 = none, 1 = present) (Breynaert et al. 2016, 2013).

The colon was cleaned after isolation from the ileocecal junction to the anus. The macroscopic damage score was calculated depending on the extent of colonic inflammation (in cm, multiplied by 2 if severe), colonic mesenterial adhesion (0 = none, 1 = mild, 2 = severe) and colonic hyperemia (0 = none and 1 = present).

A piece of severely changed colon (approximately 1 × 1 mm^2^) was fixed in 4% formalin for histopathological evaluation and immunofluorescence staining, and the remaining part was snap-frozen for quantitative real-time polymerase chain reaction (RT-PCR). Quantitative RT-PCR and immunofluorescence analysis were performed to determine the expression of mTNF-α at the mRNA and protein levels, respectively (see Additional file 1). Histopathology was performed in paraffin embedded, 5-mm-thick transverse sections stained with hematoxylin and eosin (H&E). The histological inflammation score was calculated based on the sum of architectural changes, neutrophil infiltration, epithelial defects, mononuclear cell infiltration and goblet cell loss (Breynaert et al. 2016, 2013). Three sections per animal were evaluated, and slices were scored by an experienced pathologist who was blinded to the experimental conditions.

### Intracolic ultrasound molecular imaging of a murine model of colitis

To achieve precise imaging and lay a foundation for clinical translation, ex vivo intracolic US imaging was performed. After the mice were sacrificed, the colon was resected and cleaned. For the ex vivo colon experiment, the ends of the mouse colon were tied to plastic tubes, and normal saline was injected into the bowel lumen. Then, the colon was immersed in a normal saline bath to acquire US images. Afterwards, 1 × 10^8^ MBs (MB_TNF-α_ or MB_con_ in random order) in 1 ml of phosphate buffered saline (PBS) were injected into the lumen. After a 1-min binding period, the lumen was perfused using 20 ml of normal saline, and US images were captured (Fig. 1C). Targeted contrast-enhanced US (CEUS) imaging was performed using a clinical US imaging system (EPIQ7 digital premium ultrasound system, PHILIPS, Netherlands) with a 5-12L high-frequency linear array transducer operating with the following imaging parameters: mechanical index of 0.08, frequency of 12 MHz, and image depth of 2.5 cm. All imaging settings were kept constant throughout the imaging sessions for all colons. In each colon, US images were acquired mainly in a longitudinal plane.

### Real-time polymerase chain reaction and immunohistochemical staining for quantifying mTNF-α expression

Normal and diseased bowel segments were harvested after imaging for ex vivo analysis, including H&E staining for inflammation grading and RT-PCR and immunohistochemistry for assessment of mTNF-α expression at the protein and mRNA levels, respectively. Details for these experiments are described in the Additional file 1.

### Quantitative analysis of mTNF-α expression by ultrasound molecular imaging

After targeted US imaging, regions of interest (ROIs) were drawn around the bowel walls excluding the bowel lumen, and the signal intensity was determined by ImageJ software (National Institutes of Health, Bethesda, USA). The quantification of imaging signal intensity was achieved by the normalized intensity difference (NID [fold]), which represented the ratio of MB signal intensity after injection to baseline signal intensity. Quantification of mTNF-α expression was measured as the mean integrated optical density (IOD) under four representative fields (× 200) for each sample through Image-Pro Plus v6.0 software (Media Cybernetics Inc, Bethesda, MD) as reported previously (Zhu et al. 2008). Linear regression analyses were performed to evaluate the correlations between the expression levels of mTNF-α and targeted US imaging signal intensity by NID.

## Ex vivo human intestine experiment

### Ultrasound molecular imaging of surgical specimens from IBD patients

IBD patients who underwent surgical intestinal resection at The First Affiliated Hospital of Sun Yat-sen University from April 2019 to January 2020 were included in this study. Informed consent and approval from the Institutional Research Ethics Committee (IREC) of the First Affiliated Hospital of Sun Yat-sen University were obtained, in accordance with the rules and regulations concerning ethical issues on research use of human subjects in China.

A total of 14 intestinal specimens from 8 IBD patients (CD, n = 6; UC, n = 2) who were not exposed to anti-TNF therapy but received other medications (such as 5-aminosalicylates, corticosteroids, azathioprine, and antibiotics) were enrolled. After resection from IBD patients, the fresh intestines, including normal (at margin) and diseased areas (with a length of 2–3 cm), were preserved in UW solution (University of Wisconsin solution) (Oltean and Churchill 2014) and cleaned using normal saline. The intestines were subsequently immersed in a degassed normal saline bath, and grayscale and CEUS images were obtained. Thereafter, 1 × 10^8^ MB_TNF-α_ or MB_con_ in random order in 1 ml of normal saline was sprayed onto the intestinal mucosa, and a 1-min binding period was maintained. Then, the cells were washed three times with normal saline to remove the free MBs, and images were acquired. After that, a high-frequency pulse was applied to destroy all MBs. The intestines were immersed in normal saline for washing three times. Targeted US imaging was performed using a clinical US imaging system (EPIQ7 digital premium ultrasound system, PHILIPS, Netherlands) with a 5-12L high-frequency linear array probe operating with the following imaging parameters: mechanical index of 0.08, frequency of 12 MHz, and image depth of 4.0 cm. All intestines were imaged with consistent imaging settings.

### Quantitative analysis of mTNF-α expression

Intestinal specimens were resected for H&E staining and immunofluorescence analysis to determine the histological inflammatory activity and the expression of mTNF-α, respectively. Details regarding the performance of the H&E and immunofluorescence experiments are as described above (see Additional file 1). The histologic scoring system for CD was modified from D’Haens et al. (Regueiro et al. 2019), which consisted of 8 items for each sample, and semiquantitative evaluation was as follows: inactive, 0; mildly active, 1–5; moderately active, 6–10; and severely active, 11–14 (Additional file 1: Table S1). Histological scoring of the inflammatory severity of UC was based on the Nancy index (Marchal-Bressenot et al. 2017) and included 5 items that evaluated the lumina propria lymphoplasmacytic inflammation, neutrophilic inflammation, and ulcers to arrive at the final grade (Additional file 1: Table S2). Additionally, a linear regression analysis between the targeted US signal intensity NID and mTNF-α protein expression was conducted to confirm the accuracy of targeted TNF-α US imaging in human specimens.

## Statistical analysis

Statistical analysis was performed by two-sided Student’s t-test or one-way analysis of variance using SPSS software (version 20.0, IBM Inc.). All data are presented as the mean ± standard deviation, but the clinical characteristics of IBD patients are presented as the mean (range), and *P* ≤ 0.05 was considered statistically significant.

## Results

### Characterization of microbubbles

**Fig. 2:**
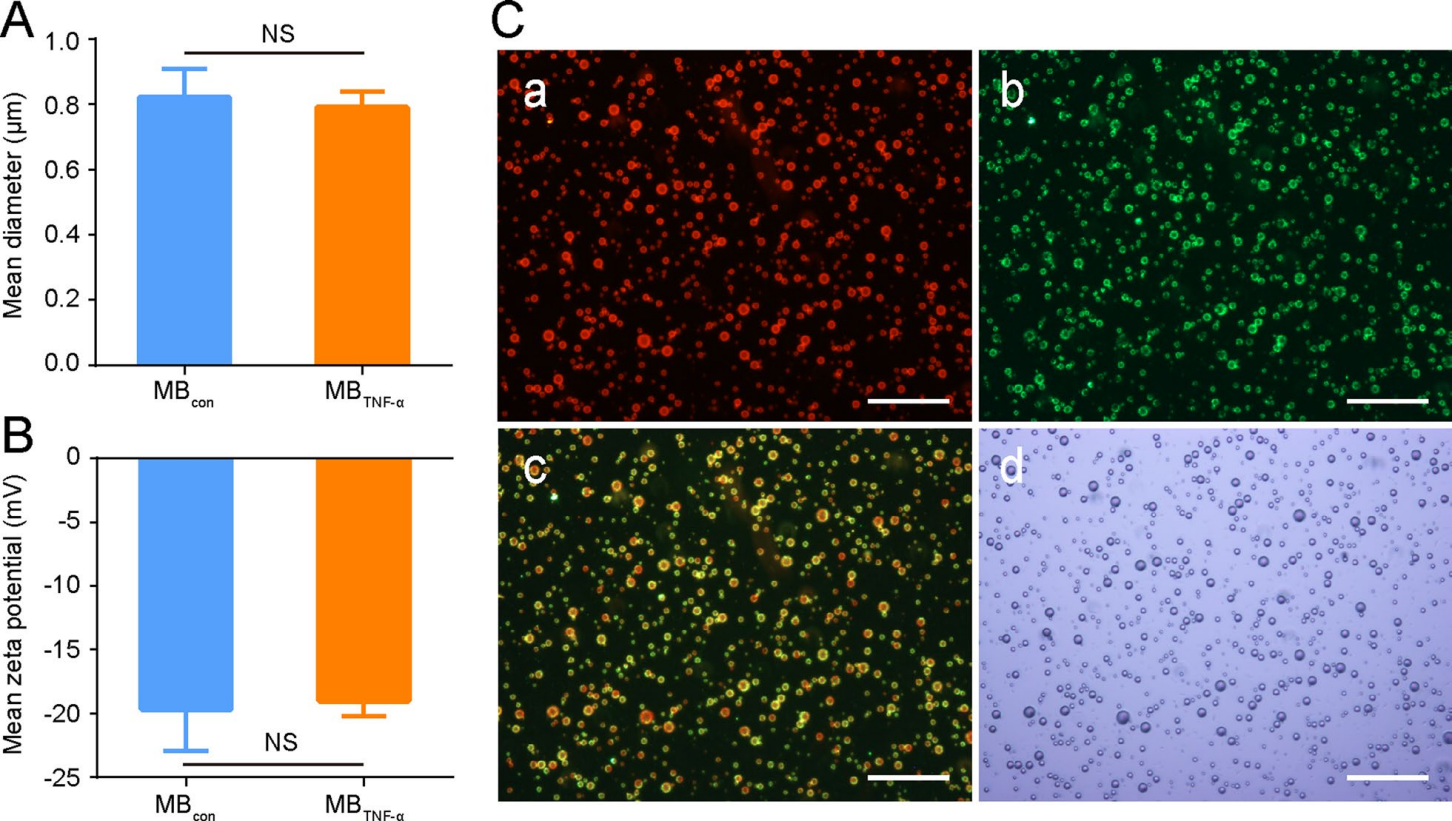
Characterization of MBs. **A** Mean diameters of MB_con_ and MB_TNF-α_. **B** Mean zeta potentials of MB_con_ and MB_TNF-α_. (NS, nonsignificant). **C** Observation of the structures of MB_TNF-α_ using inverted fluorescence microscopy (× 400, scale bar = 50 μm). **a** The lipid shells of MBs were stained with DiI. **b** Alexa Fluor 488-labeled secondary antibodies were used to locate TNF-α antibodies. **c** Merged image. **d** Bright field image

The average diameter of MB_TNF-α_ was 0.79 ± 0.05 μm, which was similar to that of MB_con_ (0.82 ± 0.09 μm, *P* = 0.742) (Fig. 2A). In addition, MB_TNF-α_ showed an approximately similar zeta potential compared to MB_con_ ([-19.63 ± 3.3] mV vs. [-19.0 ± 1.2] mV, *P* = 0.811) (Fig. 2B). These results demonstrated that the type of conjugated antibodies had no significant effect on the size and zeta potential of MBs.

The successful fabrication of MB_TNF-α_ was validated by using inverted fluorescence microscopy. As shown in Fig. 2C, the red fluorescence from DiI was encapsulated to visualize MBs, and green fluorescence from Alexa Fluor 488-labeled secondary antibody was used to trace the TNF-α antibody. The red and green fluorescence signals overlapped with each other on the merged image, which indicated the specific conjunction of TNF-α antibody to MBs. The MB concentration was [3.11 ± 0.67]×10^8^/ml. The number of antibodies on per MB was 296 ± 13.

### Evaluation of colonic inflammation and mTNF-α expression

The relative body weight change is shown in Additional file 1: Figure S1A, and a significant difference was observed at day 8 between TNBS-treated and control mice ([95.15% ± 1.97%] vs. [106.85% ± 3.19%], *P* < 0.001). The DAI and macroscopic damage score ([3.60 ± 1.85] and [3.90 ± 1.30], respectively) were significantly increased in TNBS-treated mice compared to those of corresponding controls (both [0.00 ± 0.00], both *P* < 0.001) (Additional file 1: Tables S3, S4). The histological inflammation score was significantly higher in TNBS-treated mice than in control mice ([8.43 ± 2.44] vs. [0.00 ± 0.00], *P* < 0.001) (Additional file 1: Figure S1C, D).

**Fig. 3:**
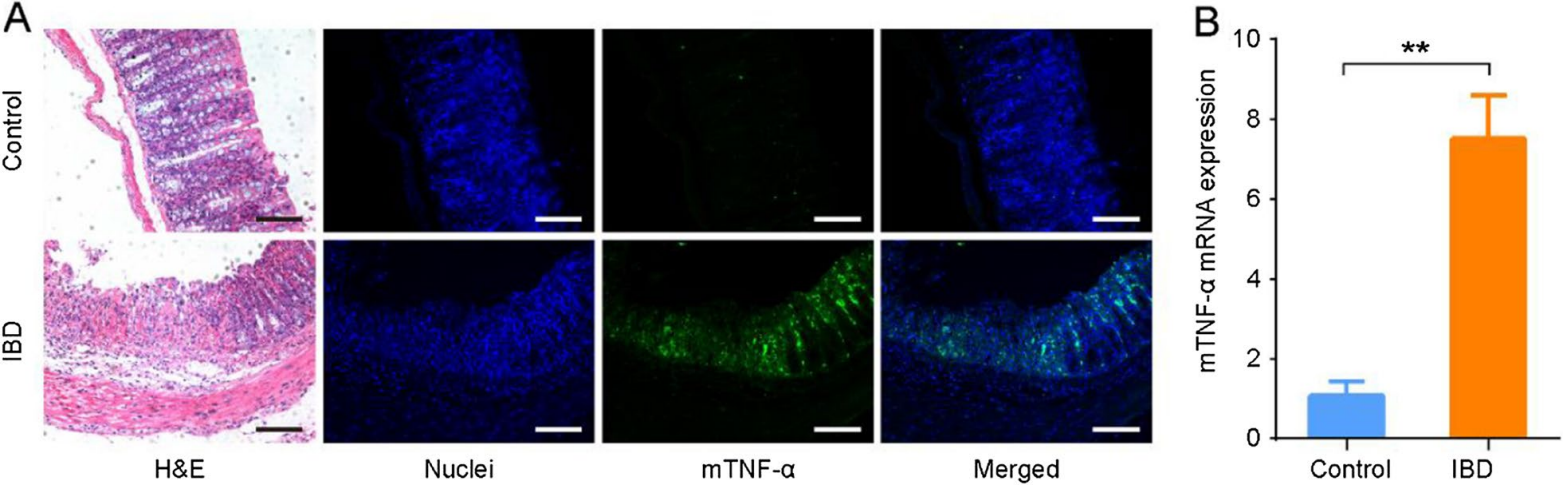
Qualitative and quantitative assessments of mTNF-α expression in TNBS-treated mouse models. **A** Representative images of H&E staining and immunofluorescence assays. The nuclei were labeled with DAPI (blue fluorescence), and mTNF-α was labeled with Alexa Fluor 488 (green fluorescence) (scale bar = 100 μm). **B** Relative mRNA expression of mTNF-α in colon tissues determined by RT-PCR. (**P = 0.001, n = 3)

Immunofluorescence analysis of control mice showed little expression of mTNF-α in the colonic mucosa, whereas the TNBS-treated colons showed a strong fluorescence intensity of mTNF-α within the local region of inflammation (Fig. 3A). RT-PCR analysis showed that mTNF-α expression significantly increased in TNBS-treated colons compared to that of controls at the mRNA level ([7.50 ± 1.11] vs. [1.06 ± 0.27],* P* = 0.001) (Fig. 3B).

### Ex vivo TNF-α-targeted US imaging and quantitative analysis of IBD mice

**Fig. 4:**
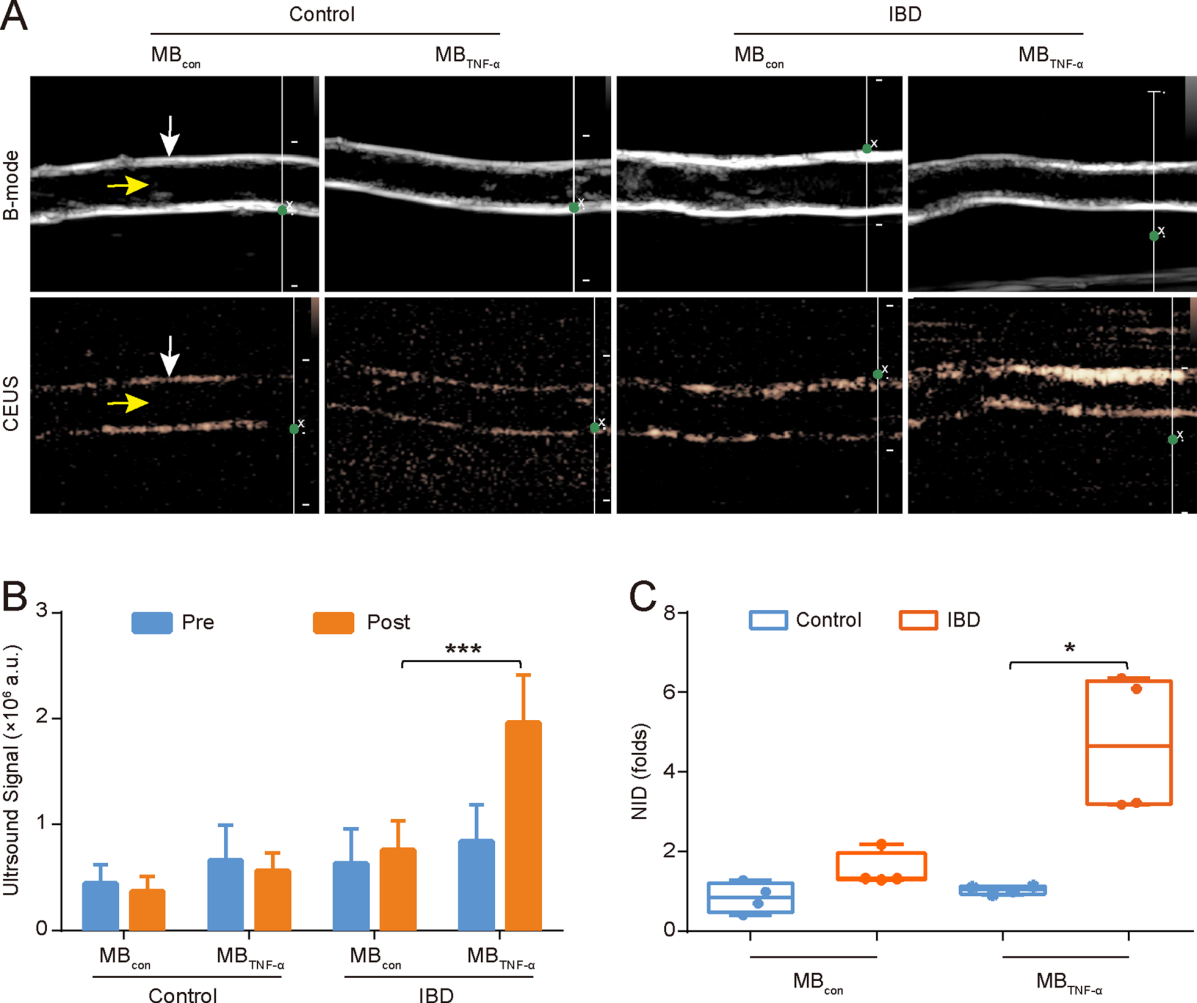
MB_TNF-α_-based ultrasound molecular imaging and quantitative analysis. **A** Representative targeted US images of MB_TNF-α_ and MB_con_ in the IBD and control groups (white arrow: intestinal wall, yellow arrow: intestinal lumen). **B** Comparison of ultrasound signals after MB administration between the IBD and control groups (***P < 0.001, n = 8). **C** The NID after MB administration (n = 4, *P = 0.024). *NID* normalized intensity differences

When treated with MB_TNF-α_, a higher targeted signal intensity was observed in the colon of colitis mice compared with that of control groups, and few signal intensities were shown in both colons of colitis and control mice after application of MB_con_ (Fig. 4A). According to quantitative analysis, colons with acute colitis showed a significantly increased US signal when treated with MB_TNF-α_ ([1.96 ± 0.45] × 10^6^ a.u.) compared to that with MB_con_ ([0.76 ± 0.28] × 10^6^ a.u., *P* < 0.001). In contrast, there was no significant difference in the ultrasound signal intensity between MB_TNF-α_ ([0.56 ± 0.21] × 10^6^ a.u.) and MB_con_ ([0.47 ± 0.13] × 10^6^ a.u., *P* = 0.242) in the control mice (Fig. 4B).

There was a significant difference in the NID between TNBS-treated (4.84 ± 1.79) and control colons (1.07 ± 0.10) after administration of MB_TNF-α_ (*P* = 0.024). However, no significant difference was observed in TNBS-treated (1.47 ± 0.37) and control colons (0.87 ± 0.39) after the injection of MB_con_ (*P* = 0.067) (Fig. 4C).

**Fig. 5:**
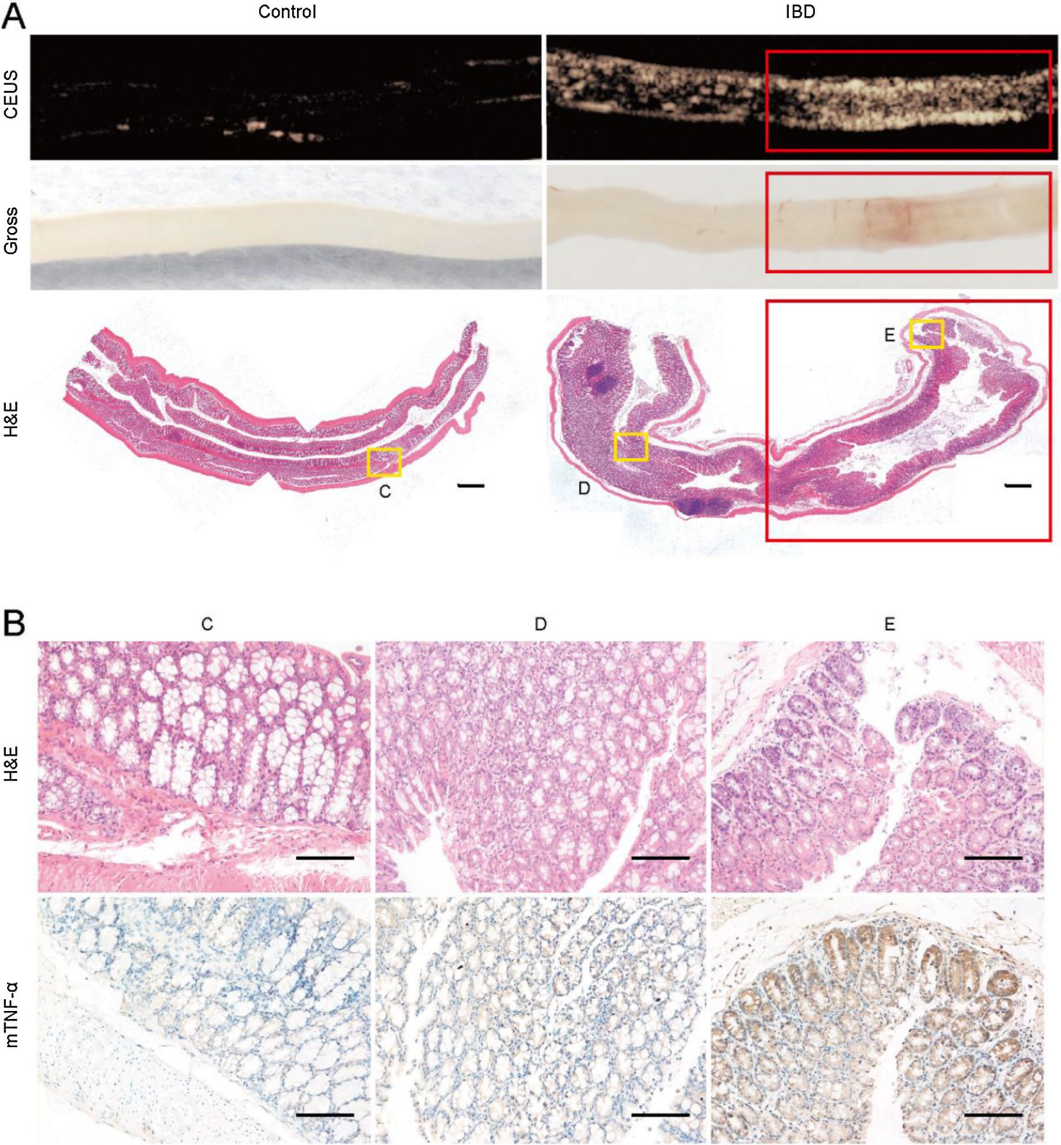
Inflammation mapping by using MB_TNF-α_. **A** Representative targeted US images of MBs for mapping inflammatory segments, gross specimens and histopathology of control and TNBS-treated mice. The red frame presents the diseased end of the colitis colon (× 40, scale bar = 50 μm). **B** H&E staining and mTNF-α expression determined by immunohistochemistry of colon tissues. **C**–**E** represent the corresponding sites framed by yellow boxes in (**A**). **C** Control mice and **D** and **E** normal and diseased segments, respectively (× 200, scale bar = 100 μm)

In addition, US mapping images of the inflammatory segment were obtained. The colon of TNBS-treated mice showed an obvious CEUS signal in the region where inflammation was evident in gross specimens and histopathology, which was consistent with mTNF-α expression confirmed by immunohistochemical analysis. A relatively weak targeted US signal and low mTNF-α expression were observed in the region with less inflammation in TNBS-treated colons. In contrast, control mice demonstrated no targeted US signal and negative expression of mTNF-α (Fig. 5).

**Fig. 6:**
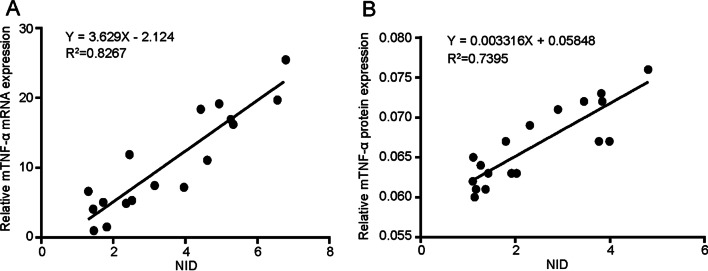
Correlation analysis between targeted TNF-α ultrasound signal normalized intensity difference (NID) and mTNF-α expression in the colons of both IBD and normal mice. The NID and mTNF-α expression had a linear correlation with a coefficient of determination of R^2^ = 0.8267 at the mRNA level (**A**, *P* < 0.001, n = 17) and R^2^ = 0.7395 at the protein level (**B**, *P* < 0.001, n = 18)

Furthermore, to confirm the accuracy of quantitative targeted US imaging, the expression of mTNF-α at the molecular level was determined by quantitative RT-PCR and immunohistochemical analysis. Linear regression equations regarding the NID and mTNF-α expression at the mRNA and protein levels were established (Fig. 6). The coefficients (R^2^ = 0.8267 at the mRNA level and R^2^ = 0.7395 at the protein level, both *P* < 0.001) represented the determined degree of NID relative to mTNF-α expression and demonstrated a favorable correlation between the targeted US signal intensity and pathological results.

### TNF-α-targeted ultrasound imaging of ex vivo human intestinal specimens

The baseline characteristics are shown in Table 1. The mean CRP was 26.77 (0.87–85.4) mg/L. All patients had accessible endoscopies, including 4 ileum lesions, 2 colon lesions and 2 involving both the ileum and colon. According to histological assessment, 3 specimens were scored as normal, and 11 specimens showed varying degrees of inflammation (mild, n = 3; moderate, n = 5; and severe, n = 3).

**Table 1:** Baseline and clinical characteristics

Characteristics	Value (n = 8)
Male/Female	5/3
Age, years (mean (range))	40.75 (16–72)
Body weight, kilograms (mean (range))	47.94 (38–55)
Disease duration, years (mean (range))	5.78 (0.03–20)
Baseline CRP, mg/L (mean (range))	26.77 (0.87–85.4)
Disease diagnosis, CD/UC	6/2
Histological activity grade (N/Mi/Mo/S)	3/3/5/3
Disease localization (ileum/colon/both)	4/2/2
Enterocutaneous or perianal fistula, n (%)	2 (25)
Previous TNF antagonist exposure	0
Concomitant medication, n (%)	
5-Aminosalicylates	3 (37.5)
Corticosteroids	2 (25)
Azathioprine	2 (25)
Antibiotics	6 (75)
Methotrexate	1 (12.5)

Histological activity grade: *N* normal, *Mi* mild inflammation, *Mo* moderate inflammation, *S* severe inflammation

**Fig. 7:**
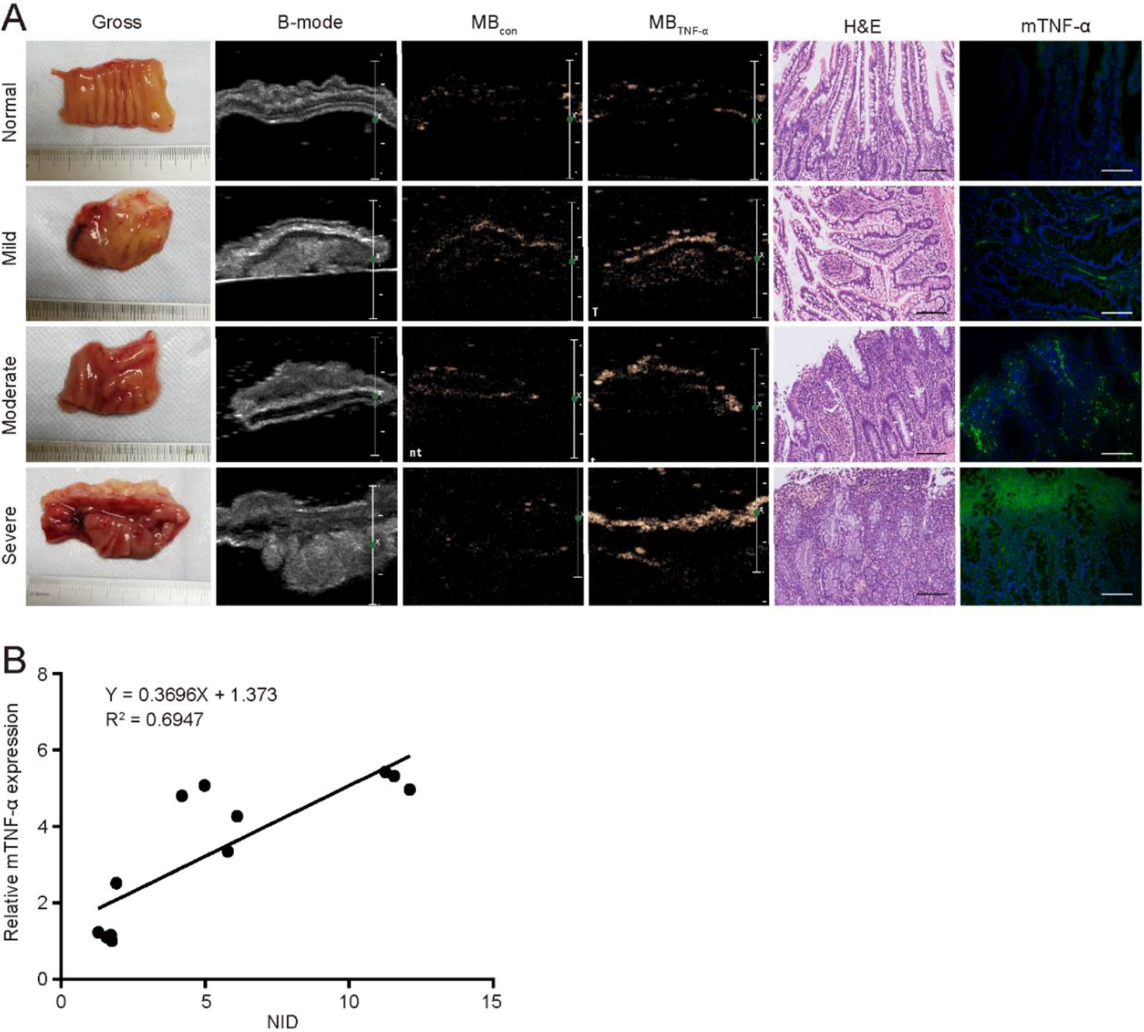
Representative images evaluating mTNF-α expression in human IBD specimens and linear regression analysis. **A** Targeted US imaging and immunofluorescence results (green fluorescence) demonstrated the weakest mTNF-α expression in normal intestines and the strongest mTNF-α expression in severe lesions compared with mild lesions. From left to right: gross specimens, B-mode US images, CEUS images after injection of MB_con_, CEUS images after injection of MB_TNF-α_, H&E staining and immunohistochemical pictures (× 200, scale bar = 100 μm). **B** The NID and mTNF-α expression at the molecular level had a linear correlation with a coefficient of determination of R^2^ = 0.6947 (*P* < 0.001). *NID* normalized intensity differences

According to targeted TNF-α US imaging results, few signal intensities were observed in normal intestines compared with diseased intestines. Among the diseased intestines, stronger signal intensities were observed in the severely inflammatory lesions than in the moderately and mildly inflammatory lesions, and the signal intensities of moderately inflammatory lesions were higher than those of mildly inflammatory lesions (Fig. 7A). Consistently, immunofluorescence analysis results demonstrated a higher expression of mTNF-α in severely inflammatory lesions than in mildly and moderately inflammatory lesions, whereas normal intestines showed the lowest expression of mTNF-α. Moreover, a linear regression equation between NID and mTNF-α expression at the molecular level was built (Y = 0.3696X + 1.373, where X and Y represent NID and mTNF-α expression, respectively, *P* < 0.001) (Fig. 7B). The coefficients (R^2^ = 0.6947) represented the feasibility of targeted TNF-α US imaging in quantitatively assessing mTNF-α expression in the intestinal mucosa of IBD patients.

## Discussion

To the best of our knowledge, this is the first study to date to use USMI in ex vivo intestinal specimens from a murine model of colitis and IBD patients to evaluate the expression of mTNF-α. In this study, a TNF-α-targeted US contrast agent, MB_TNF-α_, was developed, and ex vivo intracolic USMI experiments using MB_TNF-α_ were successfully conducted to accurately quantify mTNF-α expression in colons excised from a murine model of colitis. The targeted US signal intensities and the inflammatory lesions were topographically coupled in colons, indicating its potential application in inflammation detection. Furthermore, when using MB_TNF-α_ on ex vivo intestinal specimens of IBD patients, the feasibility of MB_TNF-α_ for assessing mTNF-α expression on intestinal mucosa and characterizing bowel inflammatory lesions was validated again.

Molecular imaging, including single-photon emission computer tomography (SPECT) and fluorescence molecular imaging, has emerged as an advanced approach for predicting the therapeutic efficacy of anti-TNF therapy in IBD with high specificity at the cellular and subcellular levels(Atreya et al. 2014; Brande et al. 2007). In the SPECT study(Brande et al. 2007), the intestinal uptake of ^99m^technetium–annexin V, which represented anti-TNF-induced apoptosis, correlated with the clinical benefit of anti-TNF treatment and might have potential for predicting responses. Nevertheless, SPECT was unsuitable for dynamic monitoring of CD patients due to radiation exposure. Another study(Atreya et al. 2014) in which CLE with fluorescent anti-TNF antibody was used to visualize transmembrane TNF-α-expressing cells in CD patients showed that patients with high expression of mTNF-α had higher response rates (92%) than those with low expression (15%) after anti-TNF therapy. However, the restricted field of view and inability to visualize the whole intestines limit the use of CLE in IBD.

USMI, which possesses the advantages of real-time monitoring, no radiation requirement, and wide visual fields, has been explored to potentially overcome these limitations. In several USMI studies, mucosal addressin cellular adhesion molecule-1-targeted MBs, vascular cell adhesion molecule-1-targeted MBs, and dual-selectin-targeted (targeting P- and E-selectin in vascular endothelium) MBs were reported to be able to assess inflammatory severity in IBD models(Wang et al. 2013; Tlaxca et al. 2013; Bachmann et al. 2006). However, these MBs were injected intravenously and were incapable of evaluating the expression of mTNF-α. Exploring novel MBs that are less invasive and highly target TNF-α would be another promising strategy for predicting the anti-TNF-α response.

As is known, TNF-α can be generally classified into a transmembrane form (mTNF-α) and a soluble form (sTNF-α) (Zhou et al. 2015). Among them, mTNF-α is expressed in cell membrane and locates in mucosa, while sTNF-α is released to interstitial space and blood from the former by enzymatic hydrolysis (Ardestani et al. 2013). Rather than sTNF-α, mTNF-α is overexpressed in mucosal immune cells of some IBD patients, which is able to predict the therapeutic response to anti-TNF treatment (Atreya et al. 2014; Loftus and Sandborn 2001). Given that no response in IBD patients is partially due to the absence of mTNF-α^+^ cells (Kattah and Mahadevan 2015), we developed MB_TNF-α_ to quantitatively assess mTNF-α expression in the colon mucosa. We found that mouse colons with colitis showed significantly higher US signal intensity than normal controls. In ex vivo surgical specimens of mice and patients, positive correlations between the quantitative US signal intensity and pathological molecular expression of mTNF-α were shown, providing evidence that USMI with MB_TNF-α_ is a promising approach for quantifying inflammatory severity and predicting the therapeutic response to anti-TNF-α therapy. Moreover, the mapping results indicated that USMI allowed a full view to visualize the inflamed and relatively normal intestines simultaneously.

In our study, we adopted an ex vivo intracolic USMI approach by local application of MBs. Compared to conventional transabdominal US with intravenous administration of MBs, it has the following advantages. First, intravenous use of MBs was not able to penetrate the capillary endothelium to the intestinal mucosa due to their large diameter (1–4 μm) (Kiessling et al. 2012), while intracolic application of MBs permitted direct and rapid binding of targeted contrast agents with mTNF-α on the intestinal mucosa. Second, intracolic imaging demonstrated a better image quality, eliminating the influence of gas and feces in the intestine, which is appropriate for clinical demands combined with endoscopy and has considerable potential for clinical translation. Further in vivo study in large animals or human could be carried out by intracolic spray of MBs with an endoscopic ultrasound system. In contrast to intravenous injection, direct spraying of MBs would not result in dilution by the blood, and more explicit images would be obtained due to the absence of gas when water was injected into the intestine. Third, fewer MBs are needed for US imaging to visualize mTNF-α expression on the intestinal mucosa, and potential allergic reactions and negative effects on other organs were minimized since the MBs did not pass through the blood circulation. Last, unlike intravenous application of MBs involving multiple organs, intracolic USMI with local administration of MBs to the bowel lumen might gain approval easier during the scrutiny of new drug applications and may possess higher clinical translation potential.

There were several limitations in this study. First, we used ex vivo US imaging in colonic specimens of IBD mice and patients, which was unable to reflect the real physiological situation in vivo despite its great promise in clinical practice. Herein, future in vivo studies are needed to verify the predictive performance of MB_TNF-α_. Second, the sample size of IBD patients was limited in this preliminary study. A larger group size and anti-TNF therapy experiments will be performed in our future studies. Finally, a biotin-streptavidin coupling chemistry method was utilized for the synthesis of MB_TNF-α_, which was not clinically translatable due to its potential immunogenicity. Targeted contrast agents available for clinical application with favorable biosafety will be developed in the future.

## Conclusion

In summary, we proposed a MB_TNF-α_-based intracolic USMI strategy. Such noninvasive and quantitative technology provides a method to detect the expression of mTNF-α, which might open new avenues for personalized therapy in IBD.

## Supplementary Information

**Additional file 1: **Materials and Methods. **Figure S1. **Inflammation parameters after induction of acute colitis with TNBS in mice. **Table S1.** Histologic grading of biopsy specimens in Crohn’s disease. **Table S2. **Nancy index system in ulcerative colitis. **Table S3. **DAI of the control and TNBS-treated mice with acute colitis. **Table S4. **Macroscopic damage score of the control and TNBS-treated mice with acute colitis.

## Data Availability

The datasets used and/or analyzed during the current study are available from the corresponding author on reasonable request.

## Abbreviations

C_3_F_8_: Perfluoropropane
CD: Crohn’s disease
CEUS: Contrast-enhanced ultrasound
DAI: Disease activity index
DLS: Dynamic light scattering
EtOH: Ethanol
H&E: Hematoxylin and eosin
IBD: Inflammatory bowel disease
IOD: Integrated optical density
MBs: Microbubbles
MB_con_: Control microbubbles
MB_TNF-α_: TNF-α-targeted microbubbles
mTNF-α: Transmembrane tumor necrosis factor alpha
NID: Normalized intensity difference
PBS: Phosphate buffered saline
ROI: Region of interest
RT-PCR: Real-time polymerase chain reaction
SPECT: Single-photon emission computer tomography
sTNF-α: Soluble tumor necrosis factor alpha
TNBS: 2,4,6-Trinitrobenzenesulfonic acid
TNF-α: Tumor necrosis factor alpha
TNFR: Tumor necrosis factor alpha receptor
UC: Ulcerative colitis
US: Ultrasound
USMI: Ultrasound molecular imaging
