# Supplementary material for: Intracolic ultrasound molecular imaging: a novel method for assessing colonic tumor necrosis factor-α expression in inflammatory bowel disease
Source: Mol Med. 2021 Sep 23;27:119. doi: 10.1186/s10020-021-00379-z (PMC8461918; doi:10.1186/s10020-021-00379-z)
Supplement: Supplementary file 1 — Additional file 1: Materials and Methods. Figure S1. Inflammation parameters after induction of acute colitis with TNBS in mice. Table S1. Histologic grading of biopsy specimens in Crohn’s disease. Table S2. Nancy index system in ulcerative colitis. Table S3. DAI of the control and TNBS-treated mice with acute colitis. Table S4. Macroscopic damage score of the control and TNBS-treated mice with acute colitis. [file 10020_2021_379_MOESM1_ESM.docx]

**Intracolic ultrasound molecular imaging****: A novel method for assessing colonic tumor necrosis factor-α expression in inflammatory bowel disease**

Xiaoyan Miao^1,a^, Ren Mao^2,a^, Yujia You^1,a^, Huichao Zhou^1^, Chen Qiu^1^, Xuehua Li^3^, Zhihui Chen^4^, Jie Ren^1^, Minhu Chen^2^, Ping Wang^1^, Rongqin Zheng^1, *^, Tinghui Yin^1,*^

^1^ Department of Medical Ultrasonic, Laboratory of Novel Optoacoustic (Ultrasonic) Imaging, The Third Affiliated Hospital of Sun Yat-sen University, Guangzhou 510630, China.

^2^ Department of Gastroenterology, The First Affiliated Hospital of Sun Yat-sen University, Guangzhou 510120, China.

^3^ Department of Radiology, The First Affiliated Hospital of Sun Yat-sen University, Guangzhou 510120, China.

^4^ Department of Gastrointestinal Surgery, The First Affiliated Hospital of Sun Yat-sen University, Guangzhou 510120, China.

**Supplemental Materials and Methods**

1. **Preparation of targeted MBs**

Briefly, all phospholipids (18 mg of DPPC, 3.5 mg of DSPE-PEG_2000_, 1 mg of DPPA, and 0.72 μg of biotinylated-DSPE-PEG_2000_) were dissolved in 4 ml of chloroform, which was removed by a rotary evaporator to form a thin phospholipid film. Then, the film was hydrated with 720 μg of streptavidin (Sigma-Aldrich, USA) in 4 ml of phosphate buffered saline (PBS) and maintained at 60 °C in a shaking incubator for 30 min to form biotin-avidin-liposomes. Afterwards, the 4-ml solution containing liposomes was sonicated with a sonicator until the solution became clear and then 0.5-ml volume transferred to new vials. Next, the air in the vials was exchanged with perfluoropropane (C_3_F_8_), and the liposome solution was mechanically vibrated for 45 seconds using a shaker to form MBs. The suspension was subsequently washed with PBS 3 times in a centrifugal device (3000 rpm, 2 min) to remove excess streptavidin. Approximately 1ⅹ10^8^ biotin-avidin-MBs were incubated with 4.5 μl of biotinylated TNF-α antibody (Bioss, China) or biotinylated isotype control antibody (Bioss, China) (1 mg/ml). After incubation at room temperature for 30 min, the solution was subsequently washed with PBS twice in a centrifugal device (3000 rpm, 2 min) to remove the free antibodies.

Measurements of diameters and zeta potentials were performed at 25 °C on 90 Plus/BI-MAS equipment (Brookhaven Instruments Corporation, USA). Furthermore, inverted fluorescence microscopy (LEICA DMI 4000B, Germany) was used to determine the specific conjunction of TNF-α to the surface of MBs. MBs were fluorescently labeled by the addition of DiI before liposomes were formed, and Alexa Fluor 488-labeled secondary antibodies (Cell Signaling Technology, USA, 2 mg/ml) were used to trace TNF-α antibody.

Determination of the microbubble concentration was performed by using an Automated Cell Counter (Bio-Rad, USA). Briefly, a volume of 20 μl of MB dispersion diluted 100 times in PBS was added to counting slides, and then the amount of MBs was counted automatically. The experiment was repeated three times. To measure the number of antibodies on per MB, biotinylated MBs targeted to TNF-α were fabricated as mentioned above using the FITC-labeled antibody. Afterwards, MBs were destroyed by sonication in an ultrasound bath for 5 min. Then, the fluorescence of MB samples was measured using a spectrofluorometer (Tecan Spark, Austria) with 485 nm excitation and 535 nm emission. The amount of antibody on the MB surface was calculated based on the fluorescence present in the destroyed MBs, the fluorescence and amount of the antibody initially added, and the MB concentration.

1. **Murine model of TNBS-induced acute colitis**

Male BALB/C mice (6 - 8 weeks, 19.0 ± 0.9 g) were purchased from [Guangdong Medical Laboratory Animal Center](http://www.gdmlac.com.cn/index.php?q=en) (Guangzhou, China). Brieﬂy, a small patch of skin on the back of mice was shaved with an electric razor 7 days before rectal administration, and then the mice were subcutaneously presensitized with 150 μl of 1% (w/v) TNBS (Sigma-Aldrich, USA) in a 4:1 volume ratio of acetone and olive oil. Control mice were treated with presensitization solution without TNBS. On day 7, mice were anesthetized by intraperitoneal injection of pentobarbital sodium solution (30 mg/kg). A plastic feeding tube was ﬁtted to a 1-ml syringe and ﬁlled with 2.5% TNBS (a mixture of 5% TNBS solution and absolute ethanol (EtOH) at a volume ratio of 1:1) or 50% EtOH. Then, the tube was gently inserted into the colon 4 cm proximal to the anus, and 100 μl of either solution was slowly administered into the lumen. Thereafter, the mice were kept in a vertical position for 60 s and returned to the cage. The body weight of the mice was monitored every other day after presensitization and every day after rectal TNBS or EtOH administration (day 7) until sacrifice. Mice were cohoused during the experiment.

1. **Quantitative RT-PCR analysis**

Quantitative RT-PCR analysis was carried out to determine the expression of TNF-α at the RNA level. Total RNA was harvested from approximately 20 mg of snap-frozen mouse colonic tissues using the TRIzol protocol (Invitrogen, Carlsbad, USA). First-strand cDNA was synthesized using a PrimeScript® RT reagent Kit (Takara Biotechnology, Japan). TNF-α mRNA expression was quantified by real-time RT-PCR using the 2^-△△CT^ analytical method in triplicate with a 10-μl reaction mixture containing 0.5 μl of cDNA sample, 0.5 μl of each primer, 4 μl of RNA-free water and 5 μl of FastStart TaqMan probe master reagent (Roche, Germany). The procedures were performed under the thermal cycling conditions of 50 °C/5 min; 98 °C/5 min; and 40 cycles of 95 °C/15 s and 60 °C/60 s. The mRNA level of the β-actin gene in each sample was also measured as an internal reference. The primers used were as follows: TNF-α 5’-CCCTCACACTCAGATCATCTTCT-3’ (forward), 5’-GCTACGACGTGGGCTACAG-3’ (reverse), β-actin 5’-GGCTGTATTCCCCTCCATCG-3’ (forward) and 5’-CCAGTTGGTAACAATGCCATGT-3’ (reverse).

1. **Immunofluorescence**

To confirm the expression of TNF-α on colonic mucosa, immunofluorescence analysis was performed. After deparaffinization with xylene and alcohol, sections were incubated in 10 mM citrate buffer (pH 6.0) for 10 min at 90 °C for antigen retrieval. Afterwards, the sections were blocked with 10% normal goat serum for 30 min at room temperature and then incubated with anti-TNF-α and anti-CD45 polyclonal antibodies (Abcam, England) for 1 h at 37 °C. Then, after being washed with PBS, all sections were incubated with Alexa Fluor 488-labeled secondary anti-mouse antibody for 1 h. Finally, the sections were incubated with DAPI and mounted with FluorSave reagent. Images were acquired by using inverted fluorescence microscopy.

1. **Immunohistochemical staining**

Immunohistochemical staining was performed as follows. After deparaffinization with xylene and alcohol, colon tissues were incubated in 10 mM citrate buffer (pH 6.0) for 10 min at 90 °C for antigen retrieval. H_2_O_2_ (3%) in methanol was added to the samples at 4 °C for 30 min to inactivate endogenous peroxidases. Afterwards, the sections were treated with 10% normal goat serum for 1 h at room temperature for antigen blocking. The polyclonal antibody anti-TNF-α (Abcam, England) was then added to the sections for 1 h at 37 °C. Finally, after being washed with PBS, all sections were incubated with horseradish conjugated biotinylated secondary anti-mouse antibody (Vector Laboratories, USA) for 1 h. The immunoreactivity on the tissue sections was visualized using the peroxidase substrate DAB. The nuclei were counterstained by hematoxylin.

**Supplemental figures and tables**


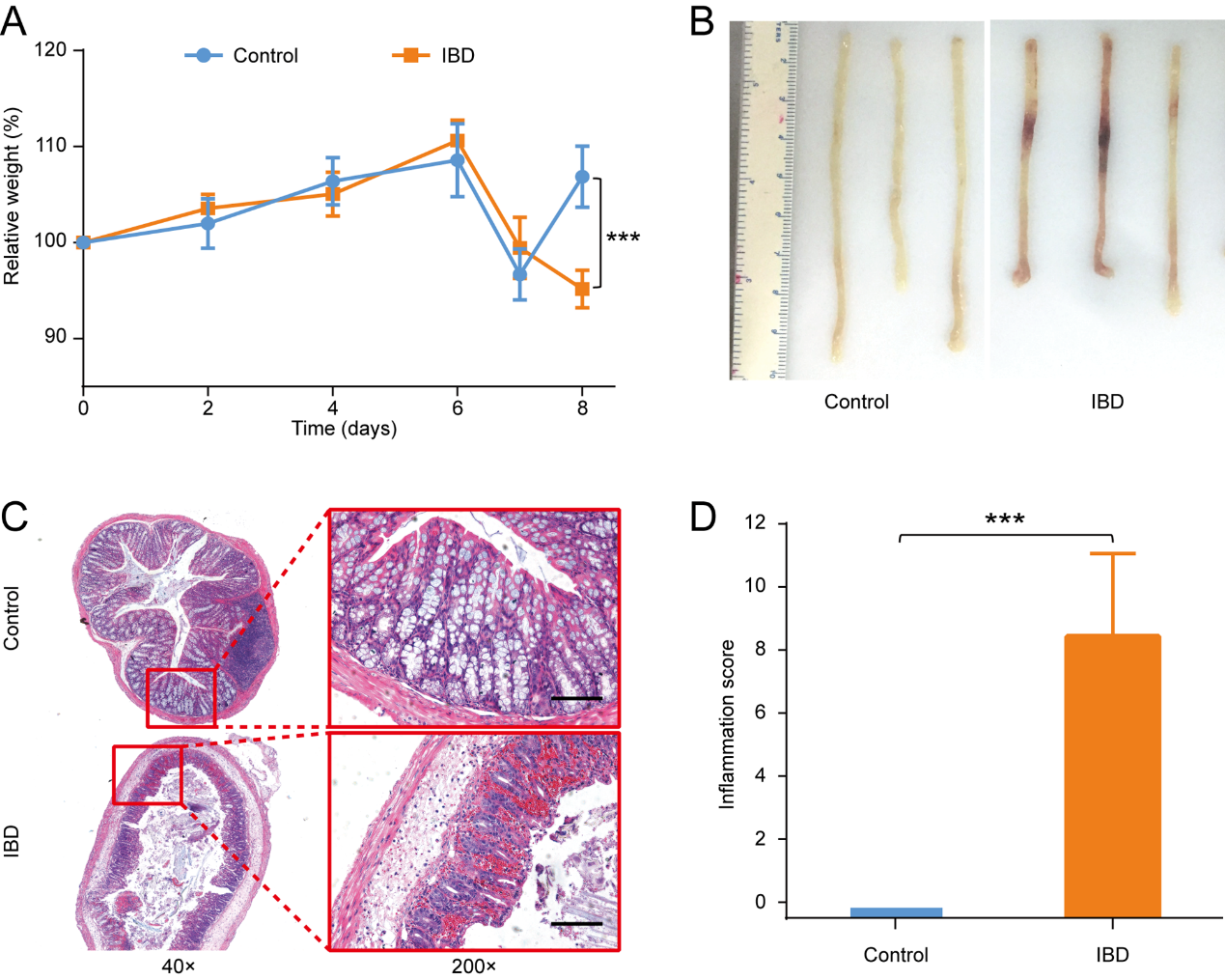


**Figure S1. Inflammation parameters after induction of acute colitis with TNBS in mice.** (A) Change in relative body weight after presensitization (**P* < 0.001, n = 10). (B) Representative images of gross specimens, (C) H&E illustrations (scale bar = 250 μm) and (D) histological inflammation scores of TNBS-treated and control mice on day 8 when the colons were excised for *ex vivo* analysis (**P*<0.001, n = 7).

**Table S1.** Histologic grading of biopsy specimens in Crohn’s disease

| Histological variable | Grading^a^ |
| --- | --- |
| Epithelial damage | 0 = normal; 1 = focal; 2 = extensive |
| Crypt architectural changes | 0 = normal; 1 = moderate (< 50%); 2 = severe (>50%) |
| Mononuclear cells in lamina propria | 0 = normal; 1 = moderate increase; 2 = severe increase |
| Polymorphonuclear cells in lamina propria | 0 = normal; 1 = moderate increase; 2 = severe increase |
| Polymorphonuclear cells in epithelium | 1 = surface epithelium; 2 = deep cryptitis; 3 = crypt abscess |
| Erosion or ulceration | 0 = no; 1 = yes |
| Granuloma | 0 = no; 1 = yes |
| Pyloric gland metaplasia | 0 = no; 1 = yes |

^a^Moderate to severe disease activity: greater than 6 to 14 (requires at least grade 1 for polymorphonuclear scores) (variables of polymorphonuclear cells in lamina propria and polymorphonuclear cells in epithelium).

**Table S2.** Nancy index system in ulcerative colitis

| Histological grading | Grade microscopic appearance |
| --- | --- |
| 0 | No histologically significant disease (no or only mild increase in chronic inflammatory cells) |
| 1 | Chronic inflammatory cell infiltrate with no acute inflammatory cell infiltrate |
| 2 | Mildly active disease |
| 3 | Moderately active disease |
| 4 | Severely active disease (ulceration) |

**Table S3.** DAI of the control and TNBS-treated mice with acute colitis.

|  | Loss of body weight | Consistency of stools | Presence of gross blood in stool | DAI |
| --- | --- | --- | --- | --- |
| Control | 0.00 ± 0.00 | 0.00 ± 0.00 | 0.00 ± 0.00 | 0.00 ± 0.00 |
| IBD | 1.00 ± 0.45 | 2.20 ± 1.40 | 0.40 ± 0.49 | 3.60 ± 1.85 |
| *P* value | < 0.001 | < 0.001 | < 0.001 | < 0.001 |

**Table S4.** Macroscopic damage score of the control and TNBS-treated mice with acute colitis.

|  | Extent of inﬂammation | Colonic mesenterial adhesion | Colonic hyperemia | Macroscopic damage score |
| --- | --- | --- | --- | --- |
| Control | 0.00 ± 0.00 | 0.00 ± 0.00 | 0.00 ± 0.00 | 0.00 ± 0.00 |
| IBD | 2.60 ± 1.02 | 0.30 ± 0.46 | 1.00 ± 0.00 | 3.90 ± 1.30 |
| *P* value | < 0.001 | < 0.001 | < 0.001 | < 0.001 |
